# Health and Economic Burden of Post-Partum *Staphylococcus aureus* Breast Abscess

**DOI:** 10.1371/journal.pone.0073155

**Published:** 2013-09-05

**Authors:** Westyn Branch-Elliman, Grace M. Lee, Toni H. Golen, Howard S. Gold, Linda M. Baldini, Sharon B. Wright

**Affiliations:** 1 Division of Infectious Diseases, Beth Israel Deaconess Medical Center, Boston, Massachusetts, United States of America; 2 Division of Infection Control/Hospital Epidemiology. Silverman Institute for Health Care Quality and Safety, Beth Israel Deaconess Medical Center, Boston, Massachusetts, United States of America; 3 Department of Population Medicine, Center for Child Health Care Studies, Harvard Pilgrim Institute and Harvard Medical School, Boston, Massachusetts, United States of America; 4 Division of Pediatric Infectious Disease and Department of Laboratory Medicine, Boston Children’s Hospital, Boston, Massachusetts, United States of America; 5 Department of Obstetrics and Gynecology, Beth Israel Deaconess Medical Center, Boston, Massachusetts, United States of America; California Department of Public Health, United States of America

## Abstract

**Objectives:**

To determine the health and economic burdens of post-partum *Staphylococcus aureus* breast abscess.

**Study design:**

We conducted a matched cohort study (N = 216) in a population of pregnant women (N = 32,770) who delivered at our center during the study period from 10/1/03–9/30/10. Data were extracted from hospital databases, or via chart review if unavailable electronically. We compared cases of *S. aureus* breast abscess to controls matched by delivery date to compare health services utilization and mean attributable medical costs in 2012 United States dollars using Medicare and hospital-based estimates. We also evaluated whether resource utilization and health care costs differed between cases with methicillin-resistant and -susceptible *S. aureus* isolates.

**Results:**

Fifty-four cases of culture-confirmed post-partum *S. aureus* breast abscess were identified. Breastfeeding cessation (41%), milk fistula (11.1%) and hospital readmission (50%) occurred frequently among case patients. Breast abscess case patients had high rates of health services utilization compared to controls, including high rates of imaging and drainage procedures. The mean attributable cost of post-partum *S. aureus* breast abscess ranged from $2,340–$4,012, depending on the methods and data sources used. Mean attributable costs were not significantly higher among methicillin-resistant vs. –susceptible *S. aureus* cases.

**Conclusions:**

Post-partum *S. aureus* breast abscess is associated with worse health and economic outcomes for women and their infants, including high rates of breastfeeding cessation. Future study is needed to determine the optimal treatment and prevention of these infections.

## Introduction

Mastitis, most often caused by *Staphylococcus aureus* [1], [2], [3], occurs in approximately 20% of breastfeeding mothers and may be a precursor to the development of breast abscess [4], [5], [6], [7]. *S. aureus* is also the predominant pathogen in post-partum breast abscess [8], [9], [10], and rates of methicillin-resistant *S. aureus* (MRSA) have increased in the pregnant and post-partum population [10], [11], [12].

Although breast abscess is a serious, uncommon complication of mastitis with high morbidity [13], [14], health services utilized by patients with these infections have been poorly characterized. Neither the clinical outcomes, such as milk fistula formation and rates of breastfeeding cessation, nor the attributable medical costs of these infections are known. Further, it is unknown if the emergence of MRSA has worsened outcomes and increased health services utilization and medical cost.

The aims of this study were to: (1) characterize the health and economic outcomes of patients with post-partum *S. aureus* breast abscess, such as breastfeeding cessation, development of milk fistulae, rates of adverse reactions to antibiotics, rates of health service utilization and medical costs and (2) explore whether costs are higher for MRSA vs. methicillin-susceptible *S. aureus* (MSSA) infections.

## Methods

### Setting

We conducted a population-based matched cohort study among post-partum women who delivered at Beth Israel Deaconess Medical Center (BIDMC), an academic tertiary care center with approximately 4,750 deliveries per year, between 10/1/2003–8/31/2010. From 10/2008–3/2010, a cluster of pulse-field type USA300–0114 MRSA infections in post-partum women and their infants who delivered at our center were identified; the majority of infections in mothers were mastitis and breast abscesses [10], [15], [16]. Cluster cases were defined as onset of MRSA post-partum breast abscess within one year after delivery.

### Case and Control Selection

Cases were defined as any woman with a culture-confirmed *S. aureus* breast abscess within one year after delivery identified via needle drainage, incision and drainage, operative intervention, or spontaneous drainage. Women with uncomplicated infectious mastitis as well as a prior history of any *S. aureus* infection were excluded from the case definition.

We selected population-based controls of pregnant women who delivered at our center (N = 32,770). Exclusion criteria for control selection included pre-partum breast abscess, neonatal demise within 24 hours of delivery, stillbirth, and culture-confirmed *S. aureus* infection at another body site. Controls were matched to cases in a 3∶1 fashion by delivery date to minimize potential bias due to secular trends in diagnosis or management of breast abscesses. Controls with a history of any prior *S. aureus* infection were excluded from the case-control analysis.

### Data Collection

Hospital databases (Infection Control, Microbiology, Obstetrics, Admission Discharge Transfer, Fiscal databases) were used to extract data for the full population-based cohort, including inpatient admissions, laboratory tests, and radiologic studies. If hospital cost data were not available, we estimated components of medical costs using the Medicare Fee schedule [17], [18].

Additional variables not available electronically were abstracted via medical record review for matched cohort study patients for up to one year after delivery or at the first visit for a subsequent pregnancy, whichever came first. Information was collected on follow-up outpatient visits to obstetrics and gynecology, internal medicine, dermatology, infectious diseases and allergy. Additional information was also collected about relevant radiographic studies (breast ultrasounds, mammograms, and breast magnetic resonance imaging), laboratory testing (complete blood counts (CBC), chemistries, wound and blood cultures), pathology (breast biopsy), central venous catheter placement, and antibiotic use and type, including use of outpatient intravenous antibiotics.

Overall hospital readmission rates were collected for the entire birth cohort. Associated adverse outcomes were extracted via chart review for matched cohort study patients only (breastfeeding cessation, milk fistula formation, and adverse effects of antibiotic use (rash, allergy)).

### Data Analysis

Health outcomes, health services utilization, and medical costs were evaluated for both the full cohort and the matched cohort. Descriptive analyses were performed using proportions, means, and medians. We compared rates of outcomes, health service utilization and costs using Wilcoxon rank-sum or Chi-squared tests as appropriate.

To maximize accuracy and generalizability, the attributable medical cost of post-partum *S. aureus* breast abscess was estimated in three ways (Table 1). First, total direct medical costs for cases and non-cases in the overall cohort were estimated from hospital fiscal databases with attributable medical costs calculated as the difference between cases and non-cases and averaged across the entire cohort. A second approach was based on estimating the difference in direct medical costs, based on hospital fiscal databases, only for those services potentially associated with *S. aureus* breast abscess (i.e., readmission, outpatient visits, laboratory testing, and radiology) for cases and non-cases in the overall cohort. Indirect medical costs, such as facility costs including overhead costs and equipment costs, were not available for analysis.

**Table 1:** Cost Estimate Methodologies.

	*Total Hospital Direct Cost*	*Partial Hospital Direct Cost*	*Medicare Cost Estimate*
**Time period**	One year	One year	One year
**Basis for estimate**	Hospital fiscal database	Hospital fiscal database	HSU x Cost per service^1^
**Inpatient direct medical costs included** 2	All	Potentially relevant	Disease-attributable
**Inpatient indirect medical costs included** 3	No	No	Yes
**Outpatient costs included** 4	All	Potentially relevant	Disease-attributable
**Outpatient indirect medical costs included**	No	No	Yes
**Medication costs included**	Some	Some	All

1 HSU = Health services utilization. Cost per service based on Medicare Fee Schedules.

2 Direct medical costs include those directly related to services provided, such as inpatient stay, outpatient office visits, laboratory and radiographic testing.

3 Indirect medical costs include facility operating costs, such as building costs, electricity costs, and costs of equipment.

4 “All” indicates that all direct medical costs, including those that may not be related to a diagnosis of post-partum breast abscess were included in the estimate of cost. “Potentially relevant” indicates that only direct medical costs related to potentially relevant services (such as visits to internal medicine, obstetrics, infectious diseases, and radiology) were included in the estimate of cost. “Disease-attributable” indicates that only services attributable to the diagnosis of post-partum breast abscess were included in the estimate of cost.

Finally, a third approach to estimating attributable total medical costs in the matched cohort study used national estimates of costs for each unit of health service utilization. The attributable costs were then calculated using the difference in the costs for cases versus controls, averaged across the matched cohort population. More specifically, each unit of service (e.g., hospitalization, procedures, laboratory and radiographic testing, outpatient visits) was multiplied by the cost of the unit of service based on the Medicare Fee Schedule and the Physician Fee Schedule [17], [18]. Laboratory costs were based on maximum Centers for Medicare and Medicaid Services reimbursement rates. Medication costs were estimated using the average wholesale price from the pharmacy Red Book [19] multiplied by the number of pills required to complete a standard antibiotic course.

Medical costs were adjusted to 2012 dollars using the medical aspect of the gross domestic product deflator, available from the United States Bureau of Labor Statistics [20]. Attributable cost was calculated by determining the mean medical cost of cases and subtracting the mean medical cost of control patients.

#### Pre-specified sub-analysis

We compared health services utilization and medical costs in case patients with MSSA to case patients with MRSA using descriptive statistics, as outlined above. A p-value of <0.05 was considered statistically significant. Data were analyzed using SAS version 9.3 (SAS Institute, Cary NC).

### Ethical Considerations

Institutional Review Board approval from BIDMC was obtained prior to data collection and analysis. Due to the retrospective study design, and acquisition of data through medical record review, waiver of informed consent was granted.

## Results

### Study Population

A total of 32,770 women delivered at our center during the study period. The mean maternal age among all women delivering at our center was 32.4 years, and 48.1% were primiparous. Baseline demographic description of the entire cohort including the 54 cases and 162 matched controls as well as the epidemic curve were previously published [10]. The cases included were 30 patients with MRSA abscess and 24 with MSSA breast abscess. The median time to diagnosis of *S. aureus* breast abscess after post-partum discharge was 34 days (Interquartile range (IQR), 24–49 days). There was no effect of MRSA or cluster period on time to clinical diagnosis.

During the cluster period (10/2008–3/2010), a total of 31 patients were diagnosed with culture-confirmed *S. aureus* breast abscess; 6 with MSSA infection and 25 with MRSA infection. The predominant MRSA strain found during the cluster period was pulsed-field type USA 300–0114 (data not shown) [15], [16].

Of the full cohort of 32,770 deliveries, 98.2% (32,188/32,770) returned to our center for any type of care during the one-year period following delivery. Among patients enrolled in the matched cohort study, 98.6% (213/216) patients received care at our facility during the one-year period after delivery.

### Outcomes

#### Matched cohort study

The readmission rate among case patients was 50%, compared to less than 2% among control patients (Table 2). The overall rate of breastfeeding cessation among breast abscess cases was 41%, and did not differ between MRSA and MSSA infections (Table 3). Data regarding breastfeeding cessation was unavailable for control patients.

**Table 2:** Health Services Utilization and Clinical Outcomes Among Women During the Year After Post-Partum Discharge: Matched Cohort Study Results.

	*Breast Abscess Cases (N = 54)*	*Controls (N = 162)*	*P-value* 1
**Outcomes**			
**Readmission Rate**	50%	1.9%	<0.0001
**Resource Utilization**			
**Total Number of Drainage Procedures (Median, IQR)**	2 (1–4)	0 (0–0)	<0.0001
**Antibiotic Utilization** 2	86.0%	5.6%	<0.0001
**Breast Surgery Consultation**	53.7%	0.6%	<0.0001
**Infectious Diseases Consultation**	35.2%	0.6%	<0.0001
**Readmission Length of Stay (Median, IQR)**	1 (0–5.5)	0 (0–0)	<0.0001
**Number of Breast Ultrasounds (Median, IQR)**	3.5 (2–6)	0 (0–0)	<0.0001
**Outpatient Office Visits** 3 **(Median, IQR)**	5 (2–7)	0 (0–0)	<0.0001

1 Wilcoxon rank-sum test, Fisher’s exact test or Chi square test used as appropriate.

2 During the one-year period following post-partum discharge. Includes oral and intravenous antibiotics.

3 Includes relevant office visits to obstetrics and gynecology (excluding routine post-partum care, preventive care, and visits for contraception), infectious diseases, breast surgery, internal medicine, and dermatology. No patients in the matched cohort study had allergy/immunology outpatient visits.

**Table 3:** Health Services Utilization and Clinical Outcomes: Comparing MRSA to MSSA Cases of Post-partum *Staphylococcus aureus* Breast Abscess During the Year after Post-Partum Discharge.

	*MRSA (N = 30)*	*MSSA (N = 24)*	*P-value* 1
**Outcomes**			
**Readmission Rate**	46.7%	54.2%	0.78
**Milk Fistula Formation**	16.7%	4.2%	0.21
**Breastfeeding Cessation** 2	50%	29.2%	0.25
**Allergic Reaction to Antibiotic** 3	16.7%	0%	0.059
**Resource Utilization**			
**Total Number of Drainage Procedures (Median, IQR)**	2.0 (1.0–4.0)	1.0 (1.0–5.0)	0.87
**Antibiotics Prescribed (Mean, 95% CI)**	2.64 (2.20–3.08)	2.09 (1.71–2.48)	0.064
**Breast Surgery Consultation**	60%	45.8%	0.41
**Infectious Diseases Consultation**	56.7%	8.33%	<0.0001
**Readmission Length of Stay, Days (Median, IQR)**	0.0 (0.0–6.5)	2.5 (0.0–4.0)	0.49
**Number of Breast Ultrasounds (Median, IQR)**	4.5 (2.0–6.0)	3 (2.0–5.5)	0.35
**Outpatient Office Visits (Median, IQR)** 4	6.0 (4.0–8.5)	3.0 (1.0–6.0)	0.0019

1 Wilcoxon rank-sum test, Chi-square, or Fisher’s Exact Test used as appropriate.

2 Data missing for 5/54 patients.

3 Includes only severe adverse allergic reactions, including hives and severe skin rash. Minor complications excluded.

4 Includes relevant office visits to obstetrics and gynecology (excluding routine post-partum care, preventive care, and visits for contraception), infectious diseases, breast surgery, internal medicine, and dermatology. No patients in the matched cohort study received allergy and immunology outpatient visits.

Among all breast abscess cases, six patients developed milk fistulae. The rate of breastfeeding cessation among milk fistula cases was high (66.7%) but not significantly different from all other breast abscess cases.

### Health Services Utilization

#### Full cohort

The overall rate of readmission among patients in the full cohort who returned to our center (N = 32,188) was 3.2%, and in cases (N = 54), 50% (p<0.0001).

#### Matched cohort study

Cases in the matched cohort study had significantly higher rates of physician visits, radiology utilization and antibiotic utilization than controls without post-partum breast abscess (Table 2). Across all cases, the median number of ultrasounds per patient was 3.5, range 0–17. Forty-one percent of cases (22/54) had greater than or equal to five breast ultrasounds. For controls, the median number of breast ultrasounds was zero. The rate of mammography was similar in case and control patients. No patients in the matched cohort study received breast magnetic resonance imaging.

The majority (75.9%) of the 54 breast abscess cases were treated with needle-guided drainage [21], [22], [23]. The median number of drainage procedures was two (interquartile range, 1.0–4.0), with a maximum of 15 drainage procedures in one patient. Thirteen (24%) required greater than or equal to five drainage procedures. Six (11%) had surgical incision and drainage; one occurred in the operating room. 14.8% of case patients (8/54) had spontaneous abscess drainage.

Antibiotic utilization was available for 92.5% (50/54) case patients. Ninety-eight percent (49/50) of case patients received antimicrobial therapy. In total, 16 different types of antibiotics were prescribed, including two (doxycycline, linezolid) that have unsafe or unknown safety profiles in breastfeeding women [24], [25]. Trimethoprim-sulfamethoxazole was prescribed frequently during the cluster period with a high rate of MRSA infections. 79.5% (39/49) patients were initially treated with a beta-lactam antibiotic, including 60% (18/30) patients ultimately diagnosed with MRSA infection.

Among the six patients who developed milk fistulae, there was a significantly higher rate of breast surgery consultations (6/6, 100% versus 23/48, 47.9%, p = 0.025), number of outpatient physician visits [median 8.5 (IQR, 6.0–15.0) versus median 4.0 (IQR, 2.0–7.0), p = 0.016], and outpatient parenteral antibiotic utilization (2/6, 33.3% versus 1/48, 2.1%, p = 0.030). There was a trend toward increased rates of surgical incision and drainage procedures (33% versus 9.3%, p = 0.089), number of breast ultrasounds [median 4.5 (IQR, 3.0–7.0) versus 3.0 (IQR, 1.5–6.0), p = 0.29], and number of antibiotics prescribed (3.2 versus 2.4, p = 0.080) among case patients who developed milk fistulae and those who did not.

### Costs

For the entire cohort, the mean attributable cost based on total direct medical cost was $2,414 (95% CI, $1,458–$3,370) and $2,340 (95% CI, $2027–$2610) based on only including potentially relevant services (Table 4).

**Table 4:** Healthcare Costs of Post-partum *Staphylococcus aureus* Breast Abscess Compared to Non-infected Controls.

Matched Cohort (N = 216)				
	*Cases*	*Controls*	*Difference*	*P-value*
**Total Hospital Direct Costs** 1 **(Mean, 95% CI)**	$2809 (2148–3470)	$240 (150–330)	$2569 (2162–2,976)	<0.0001
**Partial Hospital Direct Costs** 2 **(Mean, 95% CI)**	$2515 (1908–3122)	$129 (73–185)	$2,386 (2,027–2,745)	<0.0001
**Costs based on health service utilization** 3 **(Mean, 95% CI)**	$4073 (3097–5049)	$61 (21–142)	$4,012 (3,443–4,581)	<0.0001
**Total Cohort (N = 32,188)**				
	***Cases***	***Controls***	***Difference***	***P-value***
**Total Hospital Direct Costs** 1 **(Mean, 95% CI)**	$2809 (2148–3470)	$395 (356–434)	$2414 (1458–3370)	<0.0001
**Partial Hospital Direct Costs** 2 **(Mean, 95% CI)**	$2515 (1908–3122)	$175 (164–186)	$2,340 (2,070–2,610)	<0.0001
**Costs based on health service utilization** 3 **(Mean, 95% CI)**	**–**	**–**	**–**	**–**

1 Includes all direct healthcare costs based on hospital fiscal databases during the one-year period following post-partum discharge.

2 Includes only potentially relevant healthcare costs based on hospital fiscal databases, such as inpatient readmission, relevant outpatient office visits, radiology, and laboratory costs.

3 Estimated by multiplying the number of units of each relevant healthcare service by Medicare reimbursement for each service.

In the matched cohort study, the attributable cost estimate for the matched cohort study ranged from a minimum in the partial direct facility costs of $2386 (95% CI, $2,027–$2,745) to a maximum estimate based on Medicare cost of $4,012 (95% CI, $3,443–$4,581) (Table 4).

### MRSA vs. MSSA Cases

#### Outcomes

Approximately 11% (6/54) of post-partum breast abscess cases developed milk fistulae; five patients with MRSA (17%) and one patient with MSSA (4%). In total, 5/54 patients (9.3%) of patients had allergic reactions to antibiotic therapy. All allergic reactions occurred in patients with MRSA infection (5/30, 16.7%) versus MSSA infection (0/24, 0%), however, this difference did not reach statistical significance (p = 0.06) (Table 3). Allergic reactions to antibiotic therapy were primarily due to the use of trimethoprim-sulfamethoxazole and vancomycin in patients with MRSA.

#### Health services utilization

Health services utilization was similar among case patients with MRSA and MSSA breast abscesses (Table 3); however, MRSA cases had significantly more outpatient visits (median 6.0 versus 3.0) and a higher proportion of infectious diseases consultations (57% versus 8.3%, p<0.0001). There were no significant differences in readmission rates or duration of readmissions between MRSA and MSSA cases. Among MRSA cases, there was a trend toward a higher rate of outpatient parenteral antibiotic therapy (10% versus 0%) and number of different antibiotics prescribed (2.6 versus 2.1); however, these differences were not statistically significant.

#### Costs

Despite an increase in both physician visits and antimicrobial usage, there was no significant cost difference between patients with MRSA infection and those with MSSA infection, or between patients who had milk fistulae compared to those who did not (Table 5). Attributable costs were similar regardless of methodology used.

**Table 5:** Attributable Healthcare Costs of MRSA Cases Compared to MSSA Cases.

	*MRSA (N = 30) v. MSSA (N = 24)*	*P-value*
**Total Hospital Direct Costs** 1 **(Mean, 95% CI)**	$507 (−818, 1842)	0.45
**Partial Hospital Direct Costs** 2 **(Mean, 95% CI)**	$ 806 (−408, 2020)	0.19
**Medicare Estimate of Costs** 3 **(Mean, 95% CI)**	$-148 (−212, 1812)	0.88

1 Includes all direct healthcare costs during the one-year period following post-partum discharge.

2 Includes only potentially relevant healthcare costs, such as inpatient readmission, relevant outpatient office visits, radiology, and laboratory costs.

3 Estimated by multiplying the number of units of each relevant healthcare service by the Medicare reimbursement level for that service.

## Discussion

Our study is the first to comprehensively evaluate the outcomes and health and economic burdens of post-partum *S. aureus* breast abscess. In general, we found that the consequences of this infection in a generally healthy population are substantial.

According to ambulatory medical records, many women who developed post-partum breast abscess chose to stop breastfeeding due to their infection (41%). In addition, a high proportion of patients with post-partum *S. aureus* breast abscess required inpatient readmission (50%) and treatment with intravenous antibiotics. Patients with MRSA breast abscess showed a trend towards increased allergic reactions to antibiotic therapy due to the prevalent use in this group of trimethoprim-sulfamethoxazole and vancomycin, drugs that have been associated with among the highest estimated number of emergency department visits per 10,000 outpatient prescriptions [26]. Notably, in the same study sulfonamide use was associated with a significantly higher rate of moderate-to-severe allergic reactions, compared with all other antibiotic classes combined (4.3% [95% CI, 2.9%–5.8%] vs. 1.9% [95% CI, 1.5%–2.3%]) [26].

In the BIDMC cohort, the majority of patients received ultrasound-guided drainage for definitive management of their infection rather than open drainage. Multiple previous studies have demonstrated the safety and efficacy of ultrasound-guided needle drainage in the management of breast abscess [21], [22], [27], [28], [29], and that MRSA infections can be successfully treated with needle drainage [14]. Few patients (6/45) in our study required surgical incision and drainage for management, and only one required incision and drainage in the operating room, which is similar to rates in previous studies [23], [30]. A significant proportion of patients in our study required five or more drainage procedures, and some up to 17 prior to resolution, which is higher than has been found in other studies [23], [30], [31]. There was no association between MRSA infection and requirement for additional drainage procedures, which is consistent with findings in other investigations [14].

We found no association between surgical incision and milk fistula formation. In fact, five of the six patients who developed milk fistulae underwent ultrasound-guided needle aspiration of their abscesses only; prior work has demonstrated that milk fistulae occur rarely in this setting [29], [32], [33].

The attributable medical cost of post-partum *S. aureus* breast abscess was high, and in the same range as other post-partum infections, including surgical site infection after Cesarean delivery ($3761, 95% CI, $3309–$4275) and post-partum endometritis ($4216, 95% CI, $3710–$4792). Medical costs were also similar to surgical site infections following breast surgery ($4967, 95% CI, $3447–$6719), all adjusted to 2012 dollars [34], [35].

To estimate medical cost, we utilized multiple methods, including an evaluation of total hospital direct cost, partial hospital direct cost, and a Medicare-based cost estimation to improve the accuracy and generalizability of our findings; all methods yielded similar results. The hospital-based estimates of cost may have been lower than the Medicare cost estimate because indirect hospital costs were not available for inclusion in the analysis (Table 1).

Interestingly, despite the fact that MRSA cases had significantly more outpatient visits and a higher proportion of infectious diseases consultations, as well as trends toward a higher rate of outpatient parenteral antibiotic therapy and allergic reactions to antibiotic treatment, we found no increase in cost associated with MRSA breast abscess when compared to MSSA infection, perhaps due to limited power to detect a difference between the two groups. Alternatively, outpatient management of these infections may have averted more costly readmissions or procedures [36]. MRSA patients and MSSA patients did not differ significantly in rates of readmission and readmission length of stay. Our results should be viewed in the context of published data on other types of *S. aureus* infections that have yielded inconsistent results as to the importance of methicillin-resistance in changing economic costs [37], [38], [39]; [40].

### Limitations

All data were collected from a single large birth cohort at a tertiary academic referral center and thus included patients with the most severe complications of post-partum breast abscess, such as milk fistulae. Thus, our experiences and cost analysis may not be generalizable to all practices or settings. To improve the generalizability of our findings, we used multiple methods to estimate attributable medical costs, including an evaluation of total and partial direct hospital costs, as well as an analysis using Medicare estimates. Medicare costs are not directly applicable to a post-partum population; however, Medicare reimbursement has been used in many analyses of healthcare costs [17], [18]. Although practices may have changed during the study period, all cases were matched to contemporary controls, which should control for changes in hospital practice.

The patients included in our study were all women of childbearing age, a traditionally young and healthy population. Our prior work demonstrated that the effect of maternal age on risk of post-partum breast abscess in the BIDMC cohort was very small (OR 1.08 per year), therefore, we did not control for age as a potential confounder in medical cost in this generally young and healthy population [10].

During our study period, there was a large cluster of healthcare-associated, community-onset infections with MRSA USA300-0114. As there was no significant difference in overall health services utilization or medical cost between MRSA and MSSA cases, this likely did not affect our overall findings. However, our results may not be reflective of all MRSA infections, but USA300-0114 MRSA infections in particular.

Additionally, although 98.2% of the patients evaluated in our study followed up at our center, we did not have complete follow up data on the entire cohort. It is therefore possible that some health services utilization and costs were not included. Further, in our evaluation of total and partial direct costs, we only included patients with follow up at our center.

## Conclusions

Post-partum *S. aureus* breast abscess frequently led to breastfeeding cessation and was associated with additional poor patient outcomes, high health services utilization, and significant attributable medical costs; the economic burden was similar for MRSA and MSSA infections.

Prevention efforts should be focused on prevention of all types of *S. aureus* breast abscess – not just MRSA. Further investigation is warranted to determine the optimal means of preventing *S. aureus* breast infections during the post-partum period.
